# Climate change‐induced distributional change of medicinal and aromatic plants in the Nepal Himalaya

**DOI:** 10.1002/ece3.9204

**Published:** 2022-08-15

**Authors:** Uttam B. Shrestha, Pramod Lamsal, Suresh K. Ghimire, Bharat B. Shrestha, Sajita Dhakal, Sujata Shrestha, Kishor Atreya

**Affiliations:** ^1^ Global Institute for Interdisciplinary Studies Kathmandu Nepal; ^2^ Himalayan Geo‐En Pvt. Ltd. Kathmandu Nepal; ^3^ Central Department of Botany Tribhuvan University Kathmandu Nepal; ^4^ National Herbarium and Plant Laboratories Lalitpur Nepal; ^5^ School of Forestry and Natural Resource Management, Institute of Forestry, Tribhuvan University Kathmandu Nepal

**Keywords:** medicinal and aromatic plants, Nepal, niche modeling, species distribution modeling

## Abstract

Medicinal and aromatic plants (MAPs) contribute to human well‐being via health and economic benefits. Nepal has recorded 2331 species of MAPs, of which around 300 species are currently under trade. Wild harvested MAPs in Nepal are under increasing pressure from overexploitation for trade and the effects of climate change and development. Despite some localized studies to examine the impact of climate change on MAPs, a consolidated understanding is lacking on how the distribution of major traded species of MAPs will change with future climate change. This study identifies the potential distribution of 29 species of MAPs in Nepal under current and future climate using an ensemble modeling and hotspot approach. Future climate change will reduce climatically suitable areas of two‐third of the studied species and decrease climatically suitable hotspots across elevation, physiography, ecoregions, federal states, and protected areas in Nepal. Reduction in climatically suitable areas for MAPs might have serious consequences for the livelihood of people that depend on the collection and trade of MAPs as well as Nepal's national economy. Therefore, it is imperative to consider the threats that future climate change may have on distribution of MAPs while designing protected areas and devising environmental conservation and climate adaptation policies.

## Graphical abstract

This figure provides climatically suitable habitats of various species of Medicinal and Aromatic Plants of Nepal along with extent of the species and hotspots of the studied species

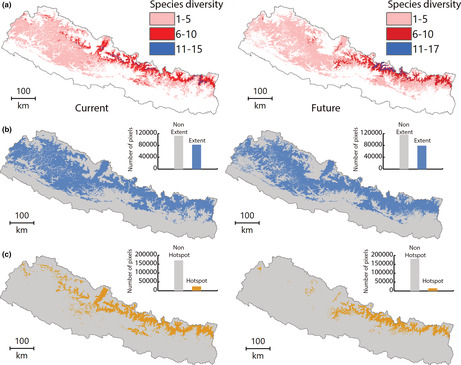

## 1 INTRODUCTION

Medicinal and aromatic plants (MAPs) contribute to human well‐being via health and economic benefits. Globally, around 28,000 plant species are currently recorded as being of medicinal use (Willis, 2017) and approximately 3000 species of them are in local, regional, and global trading systems (World Bank, 2018). More than 25% of newly marketed drugs are derived from natural products, of which majority are MAPs. For example, more than 70% of anticancer drugs are extracted from MAPs (Cragg & Newman, 2013; Newman et al., 2003). MAPs are the primary source of medicine for the majority of people living in Africa and Asia (Hamilton, 2004). With the surge of demand for natural health products and herbal drugs in recent times, the trading of MAPs is growing rapidly worldwide (Chen et al., 2016). In 2003, the annual global market for herbal medicines was estimated at US$60 billion, and by 2012, the global industry in Traditional Chinese Medicine (TCM) alone was reported to be worth US$83 billion (IPBES, 2019; Willis, 2017). MAPs are also a source of income for global rural populations through collection and sales after gathering from the uncultivated environments (Barata et al., 2016).

The Himalayan region, one of the world's biodiversity hotspots, has the highest concentration of MAP species (Kala, 2000; Olsen, 2005; Rai et al., 2000). One of the Himalayan countries, Nepal comprises 2331 recorded species of MAPs, of which around 300 species are currently under trade (Pyakurel et al., 2019; Rokaya et al., 2012). Likewise, two other Himalayan countries, China and India, have 11,146 and 7500 species of MAPs, respectively (Chen et al., 2016). The export of MAPs, including raw and processed plant products like Ayurvedic and traditional medicines produced from MAPs, in Nepal was worth around US$ 60.09 million in 2014, with an average annual export equivalent to 13.23 thousand tons (Ghimire et al., 2016). Although it shared <1% of global supplies, it contributed to approximately 5% of Nepal's GDP and 10% of the revenue collected from the forestry sector (Price, 2004). Along with a significant contribution to the national economy, MAPs also provide supplementary income and medicine for healthcare to rural households in Nepal (Larsen & Smith, 2004). In the mountainous regions of Nepal, commercial trade of wild alpine medicinal plants played an important role in the rural livelihoods, contributing on average 12% of annual household income (Olsen & Overgaard Larsen, 2003). Harvesting of a highly valued medicinal species such as the caterpillar fungus (*Ophiocordyceps sinensis*) provides even much higher income to rural households; up to 65% of the total household income of the mountain communities in Nepal (Shrestha & Bawa, 2014).

In recent decades, medicinal plants are under increasing pressure from overexploitation for trade and the effects of climate change and development (Kling, 2016). This negatively affects the large portion of the global population who rely on natural medicines and reduces the potential to identify new medicinal compounds (Hopping et al., 2018; IPBES, 2019). In the Himalaya, climate change has impacted and will likely continue to impact biodiversity and ecosystems to various degrees (Bhattacharjee et al., 2017; Shrestha et al., 2012; Shrestha et al., 2019; Xu et al., 2009). Changes in community composition, distributional range, and growth pattern of a few species, including medicinal plants, were reported or predicted in Nepal due to climate change. For example, tree lines in the high‐altitude region of the Himalaya are shifting upward (Lamsal, Kumar, & Atreya, 2017; Lamsal, Kumar, Shabani, & Atreya, 2017; Tiwari & Jha, 2018), including the growth of vegetation in sub‐nival areas of Nepal's Himalayan region (Anderson et al., 2020). The decline and increase of suitable habitats of two medicinal plant species, namely *Fritillaria cirrhosa* and *Lilium nepalense*, have been predicted (Rana et al., 2017). Conversely, the suitable habitat of the medicinal fungus, *Ophiocordyceps sinensis*, has been expected to expand in the future with climate change in Nepal (Shrestha & Bawa, 2014). A recent study showed that potentially suitable habitats of *Dactylorhiza hatagirea*, *Paris polyphylla*, and *Taxus* spp. will expand particularly toward the north of Nepal under future climate (Kunwar et al., 2021), whereas Rana et al. (2020) found decreased in potentially suitable habitats of *Paris polyphylla* and *Valerina jatamansi* while increased of *Nardostachys jatamansi*, *Neopicrorhiza scrophulariiflora*, *Aconitum spicatum*, and *Dactylorhiza hatagirea* under the future climate. Although these studies predicted the impact of climate change on the distribution of selected medicinal plants and fungi in Nepal, a consolidated understanding is lacking on how the distribution of major traded species of MAPs will change with future climate.

This study enhances the knowledge and understanding of the distribution of 29 species of MAPs in Nepal under current and future climate using an ensemble of species distribution models. We identified the current climatic envelope and estimated the future distribution of 29 species of MAPs. We further examined how the change in the distribution of medicinal plant hotspots (areas with a suitable climatic niche for the maximum number of species superimposed) will occur according to elevation, physiography, ecoregions, federal states, and protected areas. We also discussed how the future distributional change of the investigated species would affect the supply of medicinal raw materials to local people and the export industry. The results of this study will be crucial to devise conservation strategies for MAPs in Nepal, especially at a time when the conservation of MAPs from overexploitation and climate change is pertinent.

## 2 METHODOLOGY

### 2.1 Study area

**FIGURE 1:**
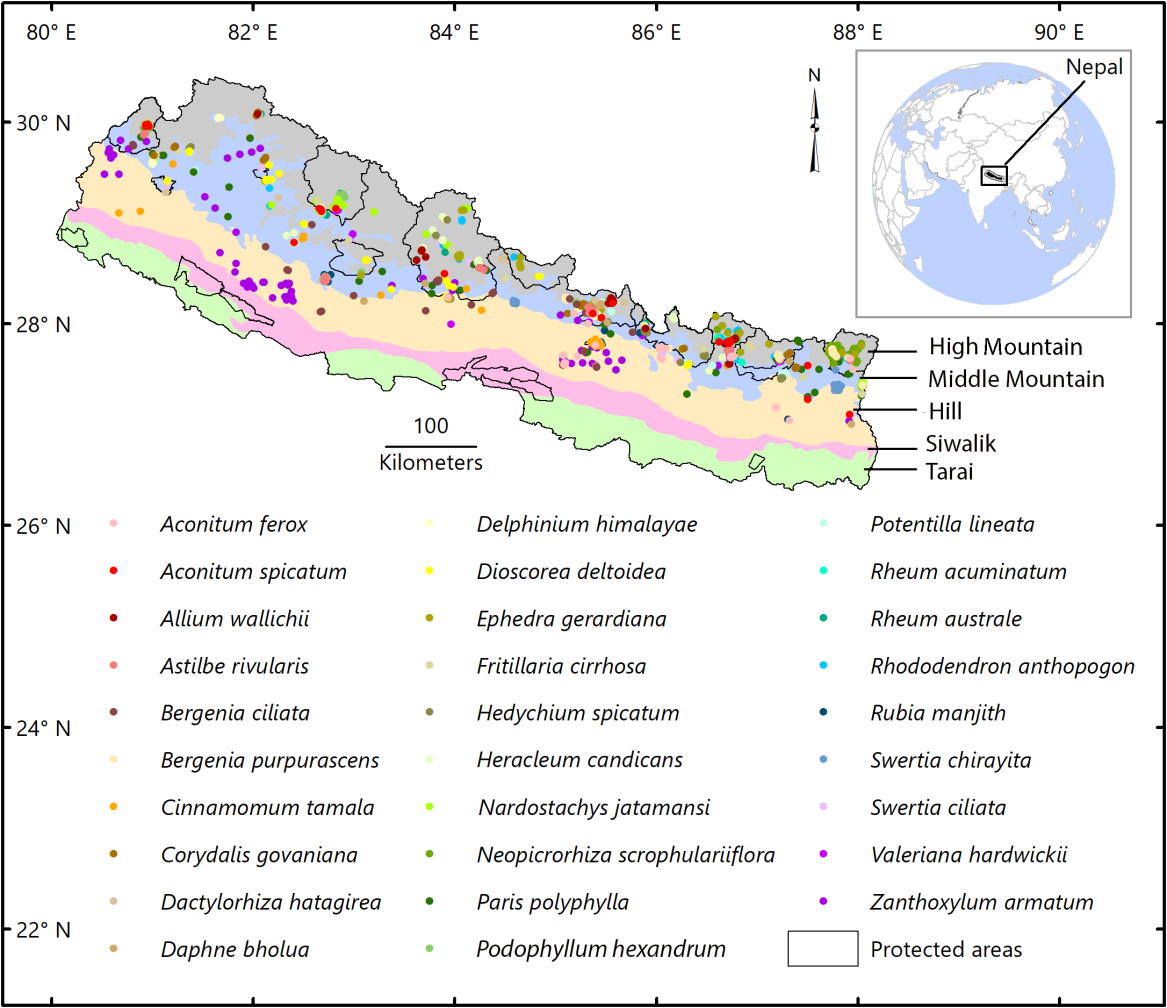
Physiographic map of Nepal showing occurrences of the 29 species of MAPs and protected areas

The study area covers the entire country of Nepal that lies at the center of the Himalaya biodiversity hotspot covering the area of 147,181 km^2^ (Figure 1). The country is divided into five physiographic zones, has sub‐tropical to alpine climates and elevation ranges from 64 to 8848 m—the Mount Everest, seven federal states, and nine Global 200 ecoregions (Olson et al., 2001). In addition, 24% of the country's land area is covered by protected areas that comprise 12 national parks, one wildlife reserve, one hunting reserve, six conservation areas, and 13 buffer zones. The country is rich in biodiversity harboring little more than 6000 species of flowering plants of which 312 are endemic to Nepal (Tiwari et al., 2019).

### 2.2 Species description and occurrence records

Twenty‐nine medicinal and aromatic plant species found in Nepal Himalaya were selected based on their wide medicinal usage, conservation status, trading value, and availability of occurrence data (Table 1). Some of the selected species are listed under the International Union for Conservation of Nature (IUCN) Red List, the Convention on International Trade in Endangered Species of Wild Fauna and Flora (CITES) appendices and national conservation lists. The selected species occur mainly in temperate, sub‐alpine and alpine zones with the lowest elevation range of 450 m a.s.l. (*Dioscorea deltoidea*) and the highest elevation range of 5000 m a.s.l. (*Nardostachys jatamansi*). Species occurrence data were gathered from field visits we made for other research projects (Ghimire 1997–2001; 2007–2018), herbaria (National Herbarium and Plant Laboratories Godavari, KATH and Tribhuvan University Central Herbarium, TUCH), Global Biodiversity Information Facility (https://www.gbif.org/ accessed: June 2019), European collections of nature history (https://www.europeana.eu/portal/en/collections/natural‐history, accessed: between April and July 2019), and published studies (Phuyal et al., 2019 for *Zanthoxylum armatum*). Utilizing Google Earth, we geocoded 30 occurrence records that only have the names of collection localities. Data collected from the various sources were compiled, and duplicates and dubious records (e.g., records that fall 1000 m out from the reported elevation range of the species) were removed. Survey biases often displayed by species distributional data could have implications for predicting species occurrence under changing environmental conditions (Dormann, 2007). Spatial autocorrelation of sampling effort between training and test data inflates the prediction accuracy (Veloz, 2009). Therefore, spatial filtering is conducted to reduce sampling biases and model over‐fitting (Boria et al., 2014; Dimson et al., 2019; Kramer‐Schadt et al., 2013). Therefore, multiple presence locations in the same grid of ~1 km^2^ spatial resolution (unit of analysis of this study) were removed and retained only one record per grid using the spatial filtering tool of SDMTOOLBOX 2.3 (Brown, 2014). Remaining 922 occurrence records were used in our ensemble modeling after removing erroneous and duplicated records. The number of occurrence locations for individual species ranged from 21 (*Allium wallichii*) to 103 (*Neopicrorhiza scrophulariiflora*).

**TABLE 1:** Overview of the 29 medicinal and aromatic plants (MAPs)

Species	Family	Local name	Life form	Elevation range (m a.s.l.)	Occurrence locations
*Aconitum ferox* Wall. ex Ser.	Ranunculaceae	Bis/Kaalo bis	PH	2100–3800	25
*Aconitum spicatum* (Brühl) Stapf	Ranunculaceae	Bish	PH	1800–4200	22
*Allium wallichii* Kunth	Amaryllidaceae	Ban lasun	PH	2400–4650	21
*Astilbe rivularis* Buch.‐Ham. ex D.Don	Saxifragaceae	Thulo/Budo okhati	PH	2000–3600	23
*Bergenia ciliata* (Haw.) Sternb.	Saxifragaceae	Paashanbed	PH	1000–3200	28
*Bergenia purpurascens* (Hook.f. & Thomson) Engl.	Saxifragaceae	Paashanbed	PH	3800–4700	28
*Cinnamomum tamala* (Buch.‐Ham.) T.Nees & Eberm.	Lauraceae	Tejpaat	T	450–2000	23
*Corydalis govaniana* Wall.	Papaveraceae	Bhutkeshee	PH	3000–4800	31
*Dactylorhiza hatagirea* (D.Don) Soó^c^, ^d^	Orchidaceae	Paanchaunle	PH	2800–3960	23
*Daphne bholua* Buch.‐Ham. ex D.Don	Thymelaeaceae	Loktaa	S	1800–3000	34
*Delphinium himalayae* Munz	Ranunculaceae	Atis	PH	3000–4500	26
*Dioscorea deltoidea* Wall. ex Griseb.^c^	Dioscoreaceae	Bhyaakur, Tarul	C	450–3100	25
*Ephedra gerardiana* Wall. ex Klotzsch & Garcke^b^	Ephedraceae	Somlataa	S	2400–4500	33
*Fritillaria cirrhosa* D.Don	Liliaceae	Kaakolee	PH	3000–4600	31
*Hedychium spicatum* Sm.	Zingiberaceae	Seto saro	PH	1500–2100	26
*Heracleum candicans* Wall. ex DC.	Apiaceae	Chimphing	PH	2200–3800	26
*Nardostachys jatamansi* Wall. ex DC.^a^ ^,^ ^c^ ^,^ ^e^	Caprifoliaceae	Bhutle, Jataamasi	PH	3200–5000	56
*Neopicrorhiza scrophulariiflora* (Pennell) D.Y.Hong	Plantaginaceae	Kutki	PH	3500–4800	103
*Paris polyphylla* Sm.^b^	Melanthiaceae	Satuwaa	PH	2000–3000	42
*Podophyllum hexandrum* Royle^c^	Berberidaceae	Laghupatra	PH	3000–4500	22
*Potentilla lineata* Trevir.	Rosaceae	Bajrandantee	PH	1600–4800	27
*Rheum acuminatum* Hook.f. & Thomson	Polygonaceae	Padamchaal	PH	3300–4200	26
*Rheum australe* D.Don	Polygonaceae	Padamchaal	PH	3200–4200	23
*Rhododendron anthopogon* D.Don	Ericaceae	Sunpaati	S	3000–4800	40
*Rubia manjith* Roxb.	Rubiaceae	Majitho	C	1200–2100	25
*Swertia chirayita* (Roxb.) H.Karst.	Gentianaceae	Chiraaito	AH	1500–2500	23
*Swertia ciliata* (G.Don) B.L.Burtt	Gentianaceae	Chiraaito	AH	2800–4000	28
*Valeriana hardwickei* Wall.	Caprifoliaceae	Nakkali jataamasi	PH	1200–4000	35
*Zanthoxylum armatum* DC.	Rutaceae	Timur	S	1100–2500	79

Abbreviations: AH, annual herb; PH, perennial herb; S, shrub; C, climber; T, tree.

^a^ Critically endangered species listed in IUCN red list (IUCN, 2012).

^b^ Vulnerable species listed in IUCN red list (IUCN, 2012).

^c^ Species listed in CITES II list.

^d^ Government of Nepal's ban on collection, use, sale, distribution, transportation, and export.

^e^ Government of Nepal's ban on export outside the country, except the processed product on permission of Department of Forest.

### 2.3 Environmental variables and model used

We downloaded 19 bioclimatic variables from the WorldClim data set (www.worldclim.org) at 30 arc sec (~1 km^2^) resolution. These bioclimatic variables were derived from monthly values of minimum, average and maximum temperature, and precipitation from 1970 to 2000 (Hijmans et al., 2005). The use of such relatively fine resolution of climate data is appropriate for regions with complicated topography, such as the Himalaya, where climatic conditions change significantly over a short distance. We analyzed a multicollinearity test among 19 bioclimatic variables and removed highly correlated variables (*r* > .70). Strong collinearity between the variables in predictive modeling could influence the overall model outcome by placing high emphasis on two or more highly correlated variables (Baldwin, 2009), resulting in misinterpretation.

The remaining seven variables: annual mean temperature (BIO1), mean diurnal range (BIO2), isothermality (BIO3), temperature annual range (BIO7), precipitation of driest month (BIO14), precipitation of warmest quarter (BIO18), and precipitation of coldest quarter (BIO19) were used as predictors for the ensemble model. We also predicted climatically suitable areas for the 29 medicinal plant species under future climatic conditions. For these predictions, projected bioclimatic variables were used for the period 2050 under the representative concentration pathway (RCP) 6.0 scenario from the Coupled Model Intercomparison Project Phase 5 (CMIP5) as presented by the Intergovernmental Panel on Climate Change (IPCC, 2013). Globally, climate model experiments have been done to produce different global climate models (GCMs) and submitted to the CMIP5 (Taylor et al., 2012). As per the IPCC (2013), RCP 6.0 is the medium future emission scenario that peaks in approximately 2040, with total radiative forcing potentially reaching +6.0 W/m^2^ (~850 ppm CO_2_ equivalent) by the end of twenty‐first century and stabilize thereafter by the employment of a range of technologies and strategies for reducing greenhouse gas emissions (IPCC, 2013).

The outputs of the GCMs for a range of periods in the twenty‐first century were used to produce gridded bioclimatic variables for future climate scenarios (Kriticos et al., 2012). We downloaded bioclimatic data of 12 global circulation models (GCMs): BCC‐CSM1‐1, CCSM4, GFDL‐ESM2G, GISS‐E2‐R, HadGEM2‐AO, HadGEM2‐ES, IPSL‐CM5A‐LR, IROC‐ESM‐CHEM, MIROCESM, MIROC5, MRI‐CGCM3, and NorESM1‐M from WorldClim (Fick & Hijmans, 2017). For more reliable outcomes, we created an ensemble of the twelve GCMs by taking average values and used the ensemble values as predictors. The multimodel ensemble average not only accounts for variability among different GCMs but also yields results superior to individual models at global and regional scales (Aguirre‐Gutiérrez et al., 2017; Murphy et al., 2004; Pierce et al., 2009). We used the same seven bioclimatic variables used for modeling current distribution to predict climatically suitable areas under predicted future climatic conditions.

### 2.4 Ensemble modeling

An ensemble modeling of species distributions involves simulations across more than one set of initial conditions, model classes, model parameters, and boundary conditions (Araújo & New, 2007). The ensemble model accounts for uncertainties in predictions of different algorithms and uses a wide range of approaches to test models (Aguirre‐Gutiérrez et al., 2017; Thuiller et al., 2009). However, a single‐algorithm modeling method, MaxEnt can produce distribution maps of comparable accuracy to ensemble models (Kaky et al., 2020). We used ensemble modeling because this consensus approach can often perform better than a single algorithm (Araújo & New, 2007; Thuiller et al., 2009). The analysis was conducted in R environment v 3.4.2 (R Core Team, 2016) using the biomod2 package (Thuiller et al., 2009). The following seven algorithms were used to produce an ensemble model: three regression methods (GAM: general additive model; GLM: general linear model; and MARS: multivariate adaptive regression splines), three machine learning methods (ANN: artificial neural network; GBM: generalized boosting model; and RF: random forest), and one classification method (CTA: classification tree analysis). These are the most widely used models in ensemble modeling (Hao et al., 2019).

Due to the unavailability of real absence data, we followed Barbet‐Massin et al. (2012) and used 5000 pseudo‐absences selected randomly for each repetition outside a buffer of 10 km from the presence points. The models were calibrated by using 70% of the occurrence points (presence and pseudo‐absence) as training data and evaluated by using the remaining 30% as testing data (Araújo et al., 2005). We repeated the process of pseudo‐absence generation three times by three evaluation runs per species, resulting in a total of 63 models per species (seven models, three evaluation runs and three pseudo‐absence selection procedures) under each climate scenario. However, the use of pseudo‐absence data might create inaccurate model performance (Liao & Chen, 2022). Therefore, the absence of real absence data is one of the limitations of this study.

We used True Skills Statistics (TSS) as an evaluation measure of model validation and predictive performance. TSS value ranges from −1 to +1 where +1 indicates a perfect agreement, and a TSS value below 0.4 indicates poor model discrimination (Allouche et al., 2006; Beaumont et al., 2016). Models with good predictive accuracy (TSS > 0.6) were used to build an ensemble from the projection outputs (Bellard et al., 2013; Gallien et al., 2012; Thuiller et al., 2009). From the 63 individual models per species, we built ensemble models using a weighted‐mean approach in which weights are awarded for each model proportionally to their evaluation metrics scores; hence, the discrimination is fair in this approach (Marmion et al., 2009). Binary maps (suitable and unsuitable) were created using the optimal threshold that maximizes the TSS score as a cutoff value, which then converted the projected occurrence probabilities during the cross‐validation procedure (Allouche et al., 2006; Liu et al., 2013; Marmion et al., 2009). This threshold is unaffected by the prevalence of species occurrence and favors sensitivity (the number of false positives) over specificity (the number of false negatives).

To identify the regions potentially suitable for the maximum number of MAPs under current and future climate, a hotspot analysis was conducted following O'Donnell et al. (2012) by aggregating maps of climatically suitable niches for all species. Maps of species diversity (cells with a higher value indicating high species diversity) and extent (cells occupied by at least a single species) and observed changes in diversity and extent of potentially suitable regions under current and future climate (e.g., Shrestha & Shrestha, 2019) were created. The aggregated map of species diversity was reclassified later using a threshold value greater than or equal to the 25th percentile of the combined values. Areas for the top 25th percentile of the combined values were considered as hotspots for the studied MAPs (Allen & Bradley, 2016; O'Donnell et al., 2012; Shrestha et al., 2019). Finally, the changes in hotspot areas for MAPs with respect to elevation, physiography, ecoregions, protected areas, and federal states were analyzed. For that analysis, ecoregion (Olson et al., 2001) and other publicly available data including digital elevation model (DEM) (https://www.usgs.gov/centers/eros/science/usgs‐eros‐archive‐digital‐elevation‐shuttle‐radar‐topography‐mission‐srtm‐1‐arc), physiography (LRMP [Land Resources Mapping Project], 1986), and protected areas (https://www.protectedplanet.net/c/world‐database‐on‐protected‐areas) were used.

## 3 RESULTS

We evaluated the performance of models by TSS performance matrix (Figure S1). The average TSS value of our models is 0.67 indicating good predictive accuracy. We excluded the models with TSS value <0.6 for building the ensemble‐models.

### 3.1 Suitable areas for individual species

**FIGURE 2:**
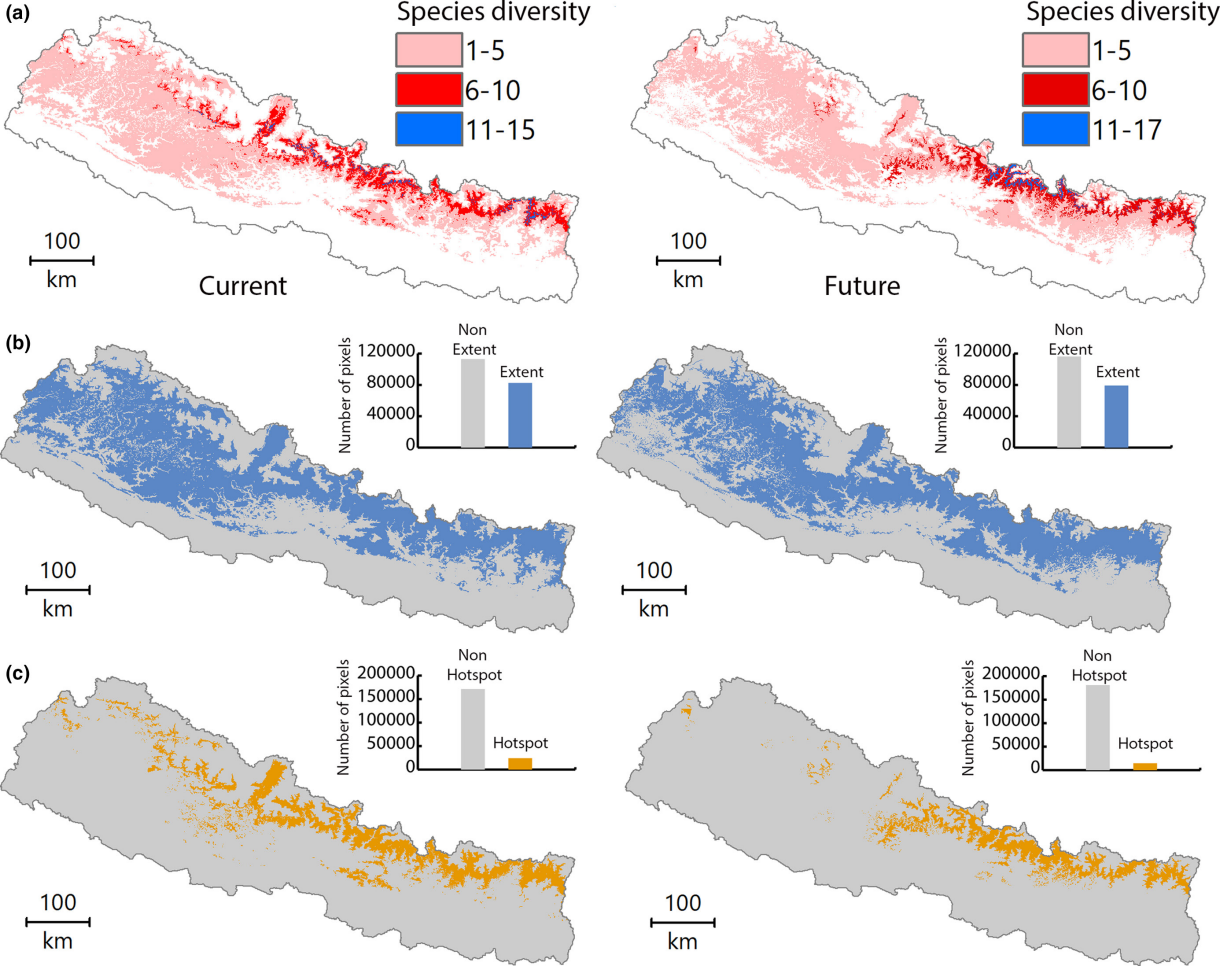
(a) Climatically suitable habitats of studied species of MAPs. (b) Extent of the studied species of MAPs. (c) Hotspots of the studied species of MAPs.

On average, 5821 km^2^ of area was predicted to be climatically suitable for individual species modeled. However, the results revealed a wide species‐specific variation in climatically suitable areas (Figure 2a). *Zanthoxylum armatum* was predicted to have the largest suitable area that is 45 times higher than the area suitable for *Swertia chirayita*, the species with the smallest suitable area (Table 2). The other species with more than 10,000 km^2^ of suitable area included *Valeriana hardwickei*, *Paris polyphylla*, and *Rhododendron anthopogon*. For eight species, including *Swertia chirayita*, *Dactylorhiza hatagirea*, *Astilbe rivularis*, and *Rubia manjith*, the suitable area was predicted to be <2000 km^2^. In the future, the average suitable area would decline by 10.4% with 5215 km^2^ being predicted to be suitable for individual species. Compared to the suitable area predicted under current climate, there will be a decline in suitable areas of 19 species (65.5%) in the future with >50% reduction predicted for the species like *Fritillaria cirrhosa* (83.8%), *Podophyllum hexandrum* (74.0%), *Delphinium himalayae* (63.3%), and *Heracleum candicans* (51.3%). Increase in suitable area was predicted for only 10 species (34.55%) with >50% increase for *Dactylorhiza hatagirea* (222%), *Hedychium spicatum* (138%), *Paris polyphylla* (81%), *Bergenia purpurascens* (73%), *Dioscorea deltoidea* (68%), and *Rheum acuminatum* (56%). Very small changes (<10%) in suitable area were predicted for eight species with the smallest area reduction being for *Ephedra gerardiana* (0.4%). Among the three species with high conservation priority, change in suitable area under future climate was predicted to be only marginal (−1.2%) for *Nardostachys jatamansi* (critically endangered and CITES II listed species) but significantly large for the remaining two species; 74% decline of *Podophyllum hexandrum* and 68% increase of *Dioscorea deltoidea*.

**TABLE 2:** Climatically suitable areas of the studied species under current and future climate

Species name	Suitable area (km^2^)		
	Under current climate	Under future climate	Change
*Aconitum ferox* Wall. ex Ser.	4664	3359	−1305
*Aconitum spicatum* (Brühl) Stapf	4010	3817	−193
*Allium wallichii* Kunth	5846	8147	2301
*Astilbe rivularis* Buch.‐Ham. ex D.Don	1706	1443	−263
*Bergenia ciliata* (Haw.) Sternb.	7992	7647	−345
*Bergenia purpurascens* (Hook.f. & Thomson) Engl.	1297	2247	950
*Cinnamomum tamala* (Buch.‐Ham.) T.Nees & Eberm.	3982	5872	1890
*Corydalis govaniana* Wall.	6762	7346	584
*Dactylorhiza hatagirea* (D.Don) Soó	1607	5169	3562
*Daphne bholua* Buch.‐Ham. ex D.Don	2972	2847	−125
*Delphinium himalayae* Munz	2648	970	−1678
*Dioscorea deltoidea* Wall. ex Griseb.	3544	5938	2394
*Ephedra gerardiana* Wall. ex Klotzsch & Garcke	6943	6915	−28
*Fritillaria cirrhosa* D.Don	8754	1416	−7338
*Hedychium spicatum* Sm.	1572	3744	2172
*Heracleum candicans* Wall. ex DC.	6603	3216	−3387
*Nardostachys jatamansi* Wall. ex DC.	8684	8581	−103
*Neopicrorhiza scrophulariiflora* (Pennell) D.Y.Hong	4652	3899	−753
*Paris polyphylla* Sm.	6660	12,047	5387
*Podophyllum hexandrum* Royle	3178	827	−2351
*Potentilla lineata* Trevir.	1967	2097	130
*Rheum acuminatum* Hook.f. & Thomson	1040	1624	584
*Rheum australe* D.Don	6650	5030	−1620
*Rhododendron anthopogon* D.Don	11,336	6891	−4445
*Rubia manjith* Roxb.	1378	1111	−267
*Swertia chirayita* (Roxb.) H.Karst.	522	319	−203
*Swertia ciliata* (G.Don) B.L.Burtt	5639	4058	−1581
*Valeriana hardwickei* Wall.	22,683	13,206	−9477
*Zanthoxylum armatum* DC.	23,515	21,455	−2060

### 3.2 Extent and hotspots

The extent of climatically suitable areas, i.e., suitable for at least one of the 29 MAP species studied, was predicted to be 57,306 km^2^ under current climate with a 4% reduction in the future (Figure 2b). About 29.5% of the current extent of suitable areas was predicted to be hotspots for the studied species that would decline to 18.5% in the future (Figure 2c). Considering the hotspot, about 40% of the current hotspot areas will be lost in the future due to climate change. The results showed that there would be an overall decline in the extent of suitable areas as well as hotspots, but the decline in hotspot area will be larger than the decline in the extent of the suitable area.

### 3.3 Hotspots in protected areas

About 52% of the current total hotspot area for the entire study area is located within 13 protected areas (Table 3). Among the protected areas, Annapurna Conservation Area was predicted to have the largest hotspot areas (2813 km^2^), followed by Gaurishanker Conservation Area (1360 km^2^) and Langtang National Park (1008 km^2^). In the future, the percentage of hotspot area within these protected areas would slightly increase and reach to about 60%. However, the hotspot area would decline in the majority (10 out of 13) of the protected areas. Among the protected areas with the large hotspot areas, the highest decline was predicted in Annapurna Conservation Area (58%).

**TABLE 3:** Climatically suitable hotspots under current and future climate

Protected areas	Hotspot areas (km^2^)		
	Current climate	Future climate	Change
Annapurna Conservation Area	2813	1179	−1634
Api Nampa Conservation Area	207	110	−97
Dhorpatan Hunting Reserve	205	5	−200
Gaurishankar Conservation Area	1360	1458	98
Kangchenjunga Conservation Area	740	589	−151
Khaptad National Park	2	0	−2
Langtang National Park	1008	1024	16
Makalu Barun National Park	863	735	−128
Manaslu Conservation Area	627	444	−183
Rara National Park	1	4	3
Sagarmatha National Park	389	301	−88
Shey‐Phoksundo National Park	462	177	−285
Shivapuri National Park	71	64	−7

### 3.4 Hotspots in global ecoregions

Across the global ecoregions, the hotspots of suitable areas are mainly concentrated in four ecoregions, namely, Eastern Himalayan alpine shrub and meadows, Eastern Himalayan subalpine conifer forests, Western Himalayan alpine shrub and meadows, and Eastern Himalayan broadleaf forests (Table 4). Each of these ecoregions had areas >2000 km^2^ that were predicted to be hotspots. In the future, hotspot area would decline in majority (7 out of 9) of the ecoregions with the highest decline occur in the Western Himalayan alpine shrub and meadows.

**TABLE 4:** Areas of climatically suitable hotspots under current and future climate

Ecoregions	Hotspot areas (km^2^)		
	Current climate	Future climate	Change
Eastern Himalayan alpine shrub and meadows	4720	3634	−1086
Eastern Himalayan broadleaf forests	2450	1824	−626
Eastern Himalayan subalpine conifer forests	3535	2569	−966
Himalayan subtropical broadleaf forests	7	12	5
Himalayan subtropical pine forests	617	783	166
Rock and Ice	903	742	−161
Western Himalayan alpine shrub and meadows	3335	374	−2961
Western Himalayan broadleaf forests	612	179	−433
Western Himalayan subalpine conifer forests	681	49	−632

### 3.5 Hotspots along elevation gradients

**FIGURE 3:**
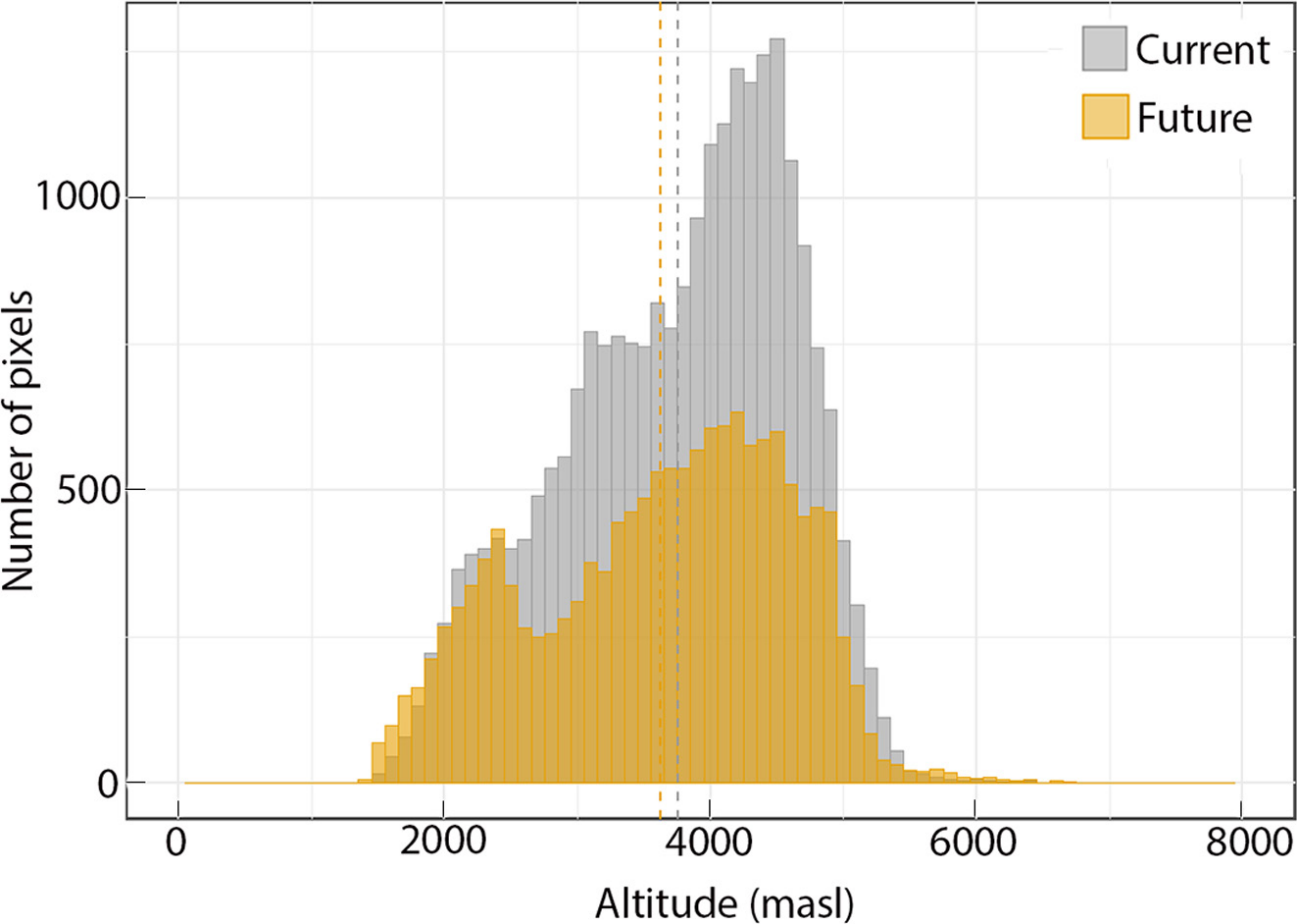
Elevation ranges of climatically suitable hotspots for MAPs under current and future climate

Distribution of hotspot areas varied significantly with elevation (Figure 3). The hotspot areas were mostly concentrated between 2000 and 5000 m a.s.l. with the largest hotspot area being predicted between 4000 and 4500 m a.s.l. For the studied species, the hotspot was not predicted below 1000 m a.s.l. In the future, a decline in hotspot area was predicted for the elevation range between 2000 and 5500 m a.s.l. and an increase in hotspot area below and above that elevation range.

**FIGURE 4:**
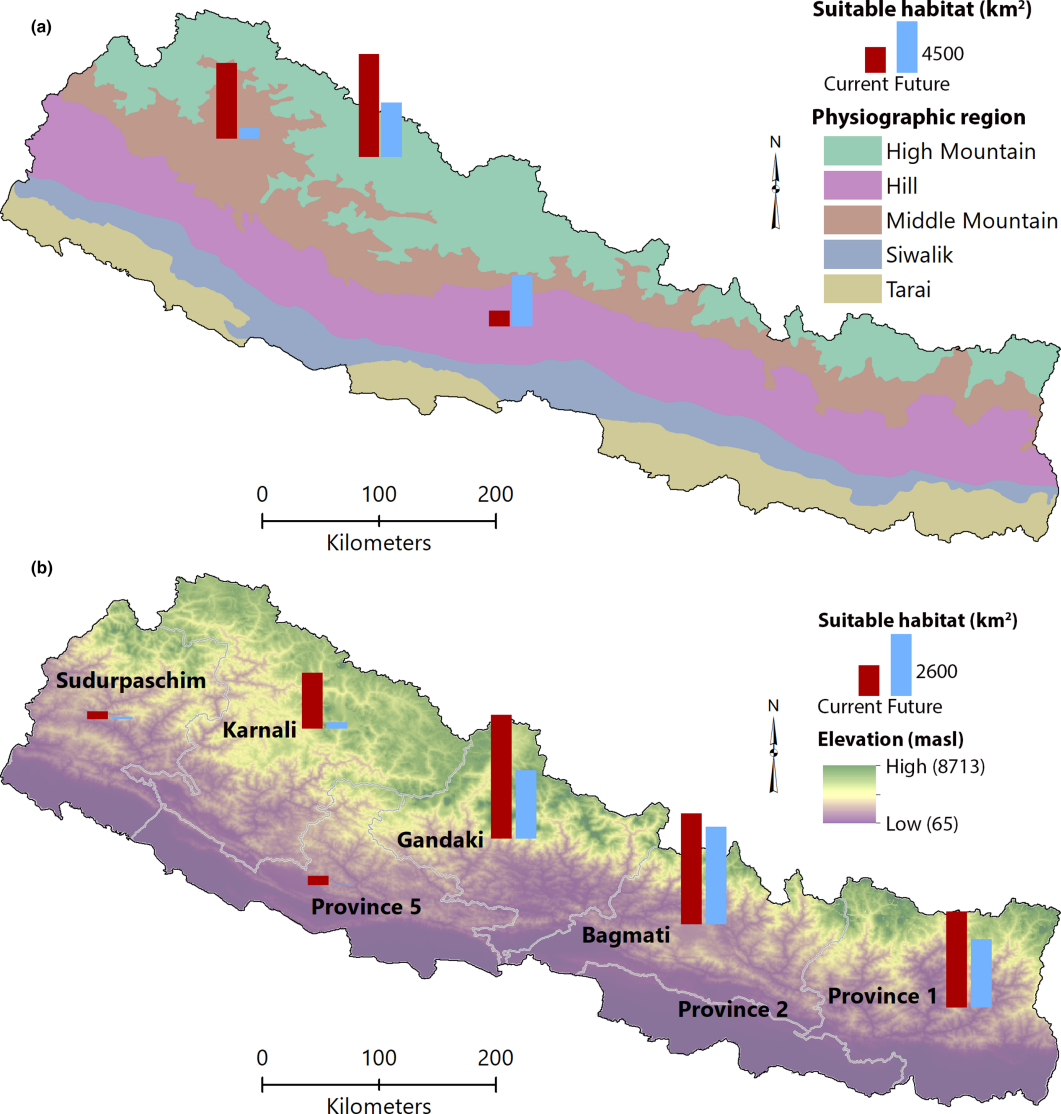
(a) Change in hotspots in physiographic zones. (b) Change in hotspots in federal states.

The hotspot areas are mainly concentrated in High Mountain regions, followed by the Middle Mountains and Hill. The High Mountain region would have the highest proportion of hotspot area but would have a 47% reduction of hotspot area under future climate scenario (Figure 4a).

### 3.6 Hotspots across federal states

There was also significant variation in the distribution of hotspot areas across the federal states (Figure 4b). The highest percentage of hotspots was predicted to lie within Gandaki province (30.6%), followed by Bagmati province (27.5%) and Province 1 (23.8%). For the studied species, there was no hotspot area in Madhesh province (Province 2) and very little hotspot area in Lumbini province (Province 5). All seven provinces would lose hotspot areas with the highest loss in Lumbini province (97.6%) and the lowest loss in Bagmati province (12.2%).

## 4 DISCUSSIONS AND CONCLUSIONS

To our knowledge, we presented a comprehensive analysis of climatically suitable areas for 29 species of wild harvested MAPs in Nepal Himalaya under current and future climate using the ensemble modeling approach for the first time. Most studies previously conducted in this region focused on single (Gajurel et al., 2014; Shrestha & Bawa, 2014) or two (Rana et al., 2017) species of MAPs in Nepal and elsewhere in the Himalaya (Li et al., 2019). This study also presented a holistic analysis of the change in climatically suitable areas across ecoregions, physiography, elevation zones, federal states, and protected areas utilizing a relatively novel approach of hotspot analysis. Therefore, the results of this study are useful for the conservation of major traded species of MAPs and future conservation planning of the protected areas considering climate change impacts on MAPs. It also contributes to our understanding of the plausible consequence of future climate change on MAPs trade as the climatic suitability of the majority of the studied species is predicted to be reduced under future climate change.

Our results of the highest concentration of climatically suitable hotspot areas of MAPs being between 2000 and 5000 m a.s.l. also corroborated with the findings of Pyakurel et al. (2019) who examined the elevational distribution of 300 species of MAPs in Nepal based on surveys among traders and secondary data. Pyakurel et al. (2019) found that more than 50% of the herbaceous medicinal plants (*n* = 119) occurred between the elevation of 1600–3600 m a.s.l. The discrepancy, especially at the maximum elevation range, is perhaps due to the selection of the MAP species primarily found in high‐elevation areas in this study. Physiographically, hill and high mountain areas were considered important for medicinal plant‐based economies because of the high proportion of occurrence of medicinal plants and associated ethnobotanical knowledge (Rokaya et al., 2012). Our results also reaffirmed a high concentration of climatically suitable hotspots of MAPs in the mountains and hills.

Nepal has already experienced warming, increased annual precipitation, and increased extreme events, including an increased number of hot days and nights and heavy precipitation (Shrestha et al., 2019). By the end of 21^st^ century, Nepal could face an increased mean annual temperature between 1.7–3.6°C and 11%–23% increased precipitation (MoFE, 2019). Climate change in Nepal has already impacted landscape phenology (Shrestha et al., 2012), the distribution of forests (Thapa et al., 2016) and tree line positions (Tiwari & Jha, 2018). Similar to scientific findings in other parts of the greater Himalayan region (e.g., Yan & Tang, 2019), our results showed both increase and decrease in climatically suitable areas for MAPs in Nepal. However, the number of species of which suitable niche would decline was higher than the number of species of which suitable niche would expand. Out of the total 29 species of MAPs, the suitable niches of 19 species (66%) would reduce under future climate. Therefore, overall, climate change will create less suitable niches for the MAP species in Nepal in the future.

In addition to climate change, excessive harvesting has caused direct threat to several species of medicinal plants in Nepal (GoN, 2018). About 44% of the traded MAPs in Nepal are herbaceous species (Pyakurel et al., 2019), and out of 29 MAP species selected in this study, 22 (76%) are herbs. Underground tuber, rhizome, bulb, or whole plants of the 24 studied of MAPs are uprooted when harvesting. The harvesting of medicinal herbs is often done before seed dispersal, thereby inhibiting natural regeneration (Ghimire et al., 2008). These excessive and detrimental harvesting techniques negatively impact the population of several species of medicinal plants (Ghimire et al., 2008; Shrestha & Bawa, 2013). Additionally, extinction risk for herbaceous plants is higher than for woody plants (Yan & Tang, 2019). However, the extinction risk depends on several factors such as harvesting pressure and parts harvested. Therefore, the existing detrimental harvesting practices combined with the predicted decline of climatically suitable areas of MAPs will pose additional threats to the MAPs population and associated trade in Nepal. This decline could affect exports of MAP species as MAPs are currently exported to 50 countries from Nepal (Ghimire et al., 2016). Furthermore, Gairola et al. (2010) and Gupta and Chaturvedi (2019) reported the possible effect of climate change on the production and composition of secondary metabolites by high‐elevation MAPs that could have direct impact on their effectiveness in curing ailments and the supply of quality raw materials to pharmaceutical industries. This could have serious effects not only for pharmaceutical industries but also for traditional healers and local communities of Nepal Himalaya as many remote mountain communities in the region still rely on MAPs for primary healthcare and income generation (Ghimire et al., 2008; Rokaya et al., 2012; Shrestha & Bawa, 2013).

Climate change results in shifting distribution of species particularly toward higher elevations (Lenoir et al., 2008; Parmesan & Yohe, 2003). However, other studies also suggested downslope shifts with climate change due to changes in precipitation and other factors (Elsen & Tingley, 2015; Tingley et al., 2012). We did not observe a monotonic shift in the elevation of the majority of the species but found heterogeneous shifts of climatically suitable hotspots below 2000 m a.s.l. and above 5000 m a.s.l. While comparing our results with other studies that include some of the species covered in this study, we found contrasting results. For example, our results of a decrease in the suitable habitats of *Nardostachys jatamansi* in Nepal contrasted with an increase in suitable niches of the same species in China (Li et al., 2019). Likewise, our result of decreased suitable niches of *Fritillaria cirrhosa* contrasted with increased suitable niches of the same species in Nepal (Rana et al., 2017). These differences might be caused by the use of other climate scenarios, modeling methods, and bioclimatic variables. Additionally, the plants may not share the same ecological niche position in China as they do in Nepal.

Our results on changes in climatically suitable hotspots in the current protected areas of Nepal have implications for the design of conservation areas in the future considering future climate change. Protected areas should be able to maintain a long‐term dynamics of biodiversity change (Pressey et al., 2007). The climatically suitable hotspots of MAPs in nine protected areas will decline under future climate change scenarios, indicating that the coverage of the existing protected areas might not be suitable to conserve highly traded MAP species in the future. Furthermore, the existing protected areas are not fully representative and failed to incorporate diverse topography, ecosystems, vegetation, flora, and fauna of the country (Shrestha et al., 2010). Therefore, ensuring adequate representation of topography, ecosystems and species is essential to improve the existing protected areas system of Nepal. Our study facilitates future conservation planning of the existing conservation areas by identifying shifts in MAPs distribution within and outside protected areas under climate change.

Our results showed that future climate change will reduce climatically suitable areas for majority of the traded MAPs in Nepal. The MAPs in Nepal have been a major contributor to traditional health care, household income, and export. Excessive and destructive harvesting practices have raised a concern toward the conservation of various species of MAPs particularly herbaceous perennial species that already have higher risk of extinction. The Government of Nepal has already listed some MAP species under the national conservation/protection list that imposes a ban on collection, trade, and export. Our results showed that climatically suitable areas of the majority of traded MAP species will be reduced with future climate change. Reduction in climatically suitable areas for MAP species might have serious consequences for the livelihood of people that depend on the collection and trade of MAPs as well as for Nepal's national economy. Therefore, we urge that attention should be paid to the threats caused by future climate change on the distribution of MAPs while designing protected areas and devising environmental conservation and climate adaptation policies.

## AUTHOR CONTRIBUTIONS

**Uttam Babu Shrestha:** Conceptualization (lead); formal analysis (lead); methodology (lead); writing – original draft (lead); writing – review and editing (lead). **Pramod Lamsal:** Data curation (equal); methodology (supporting); writing – original draft (equal). **Suresh Kumar Ghimire:** Data curation (supporting); writing – original draft (supporting); writing – review and editing (supporting). **Bharat Babu Shrestha:** Conceptualization (equal); methodology (supporting); writing – original draft (supporting); writing – review and editing (supporting). **Sajita Dhakal:** Data curation (supporting); methodology (supporting); writing – original draft (supporting). **Sujata Shrestha:** Data curation (equal); writing – original draft (supporting); writing – review and editing (equal). **Kishor Atreya:** Methodology (supporting); writing – original draft (supporting); writing – review and editing (supporting).

## Supporting information


Figure S1

## Data Availability

Upon acceptance, the data that support the findings of this study are openly available in Dryad.
