# Supplementary figures and images for: Climate change‐induced distributional change of medicinal and aromatic plants in the Nepal Himalaya
Source: Ecol Evol. 2022 Aug 15;12(8):e9204. doi: 10.1002/ece3.9204 (PMC9379350; doi:10.1002/ece3.9204)

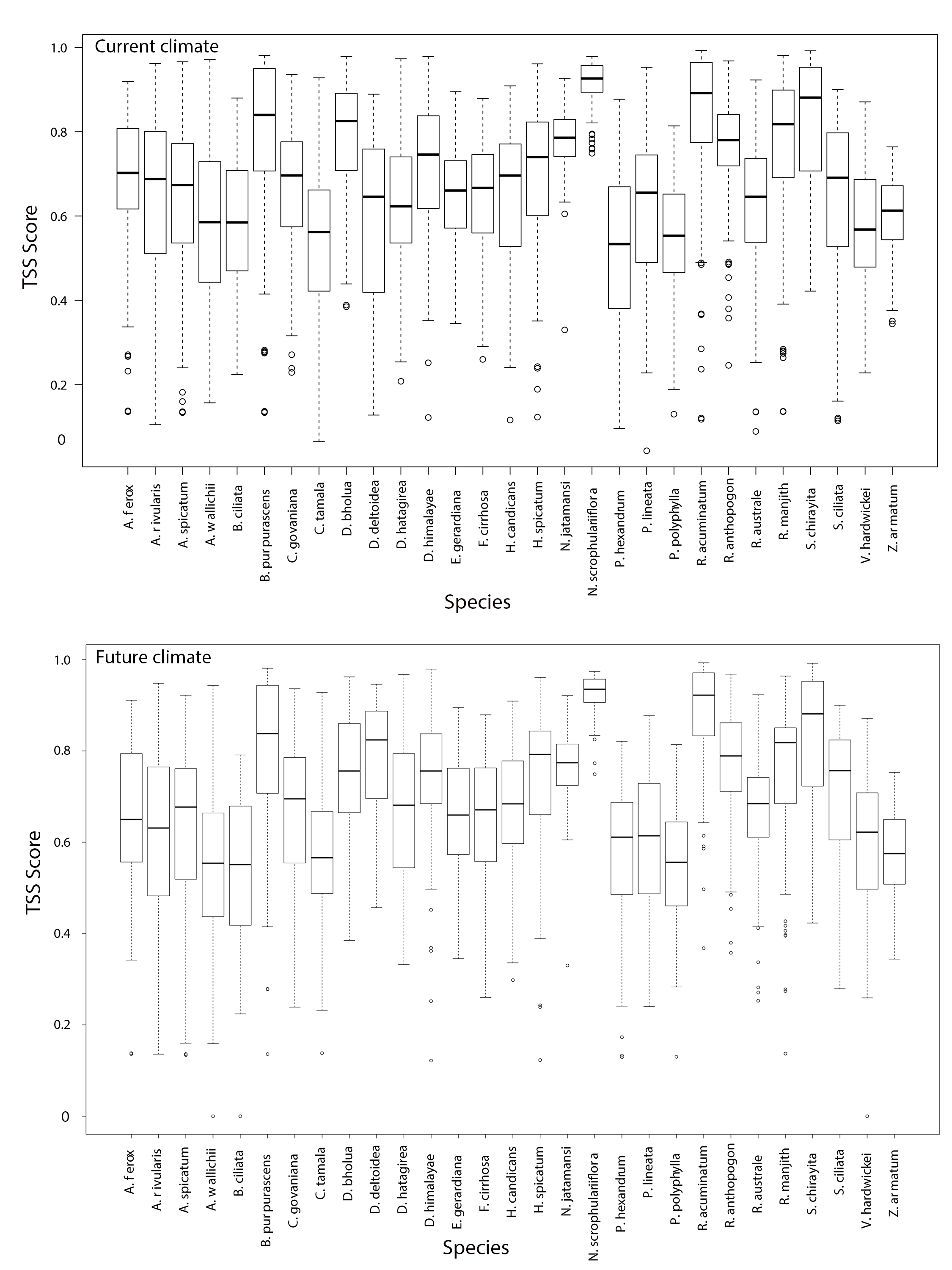

Supplement: Supplementary file 1 — Figure S1 [file ECE3-12-e9204-s001.tif]
